# DNA extraction from sweetpotato (*Ipomoea batatas*) root tissues supports routine genotyping

**DOI:** 10.1007/s11032-026-01718-w

**Published:** 2026-09-19

**Authors:** Simon Fraher, Alexander M. Sandercock, Dongyan Zhao, Tyler Slonecki, Kasia Heller-Uszynska, Andrzej Kilian, Yasmin Cummins, Vidushi Patel, Craig T. Beil, Moira J. Sheehan, G. Craig Yencho

**Affiliations:** 1 Department of Horticultural Science, North Carolina State University, Raleigh, NC 27695 USA; 2 Breeding Insight, Institute of Food and Agricultural Sciences, University of Florida, Gainesville, FL 32603 USA; 3 Diversity Arrays Technology, Australian Capital Territory, Canberra, Australia

**Keywords:** *Ipomoea batatas*, gDNA extraction, DArTag genotyping, Genomic selection, Marker assisted selection

## Abstract

Sweetpotato (*Ipomoea batatas*) breeders increasingly rely on genomic tools to enhance selection decisions. However, regrowing plants with sufficient leaf material for sampling requires several months, delaying genotyping and downstream decisions. Sampling storage root tissue would provide an earlier genotyping option, but root versus leaf tissue DNA extractions have not been compared for genotyping applications. Here, we compared genomic DNA from two storage root tissues, the cambium (“flesh”) and periderm (“skin”), and evaluated their performance against fresh leaf tissue. Root samples were taken at two storage times postharvest: 4 and 16 months. The approach produced adequate DNA, as assessed by sequencing depth and missing data rates, across all tissue types and storage times. Genotyping with a targeted 3,120 DArTag SNP panel revealed highly similar allele frequencies between root and leaf tissues (R^2^ > 0.96). Within-line dosage calls showed mean concordance of 81.3–85.5% for exact matches, increasing to 97.3–98.5% when allowing a *±* 1 dose difference. While leaf tissue had higher read depth and lower missing rates, all root tissue types exceeded the minimum 90 mean read depth recommended for hexaploid dosage calling and fell within 5% of leaf tissue missing rates. Within-root tissue comparisons did not differ significantly across tissue type or storage time. Genetic relationships in principal component analysis were consistent across tissue types, supporting repeatability. Root tissues are therefore a suitable replacement for leaf tissue in routine genotyping workflows. This methodology enables faster selection decisions, resource savings, and genotyping outside the busy growing season.

**Supplementary Information:**

The online version contains supplementary material available at https://doi.org/10.1007/s11032-026-01718-w.

## Introduction

Sweetpotato (*Ipomoea batatas* (L.) Lam.) (2n = 6x = 90) is among the world’s most important crops, and sweetpotato breeders are increasingly adopting genomic breeding approaches to facilitate earlier, better breeding decisions. Before molecular markers were available, sweetpotato breeders relied on segregation ratios and heritability to increase desirable alleles in their breeding populations (Jones 1986). Early adoption of marker-based sweetpotato breeding strategies considered several marker types. Amplified fragment length polymorphism (AFLP) markers were among the first molecular markers used in sweetpotato. AFLPs were explored for their utility in markers-assisted selection (MAS) for stem nematode (Qin et al. 2009) and southern root-knot nematode resistance (Nakayama et al. 2012), linkage map construction (Kriegner et al. 2003; Cervantes-Flores et al. 2008), and for studying genomic diversity (Zhang et al. 2000; Elameen et al. 2008). Denser, more economical marker platforms emerged with greater genome coverage. Examples include simple sequence repeat (SSR) microsatellite markers (Wang et al. 2011), and, more recently, single nucleotide polymorphisms (SNPs) (Wadl et al. 2018; Mollinari et al. 2020). In 2024, a SNP-based targeted amplicon-based genotyping technology DArTag (Diversity Array Technology; DArT) was described for sweetpotato, providing cost-effective mid-density genotyping with emphasis on genic regions for sweetpotato breeders (Zhao et al. 2024). All these approaches used DNA isolated from young sweetpotato leaf tissue, which has long been considered the tissue of choice for genotyping across crops. For molecular breeding decisions, leaf tissue is typically collected from vines produced by sweetpotato storage roots that are sprouted in greenhouses during the winter months in temperate regions – a process that requires significant labor, consumes media, occupies limited greenhouse bench space, and requires watering, fertilization, and supplemental lighting.

In the US, sweetpotato breeders use phenotypic selection to identify the best clones in harvested field trials between the months of September and November. Prior to harvest, the aboveground plant parts are typically mowed before digging the storage roots, after which these storage roots are evaluated for various traits. After harvest, selected clones are transported to curing facilities where storage roots are cured at 29 °C for 3–5 days prior to storage at 13 °C at 85% relative humidity (RH) (Edmunds et al. 2008). The storage roots remain in cold storage until the spring when they are warmed and sprouted to produce plants for genotyping, field trials, and other evaluations during the coming field season. The leaves of these emergent spring plants are typically the tissue of choice and opportunity for genotyping and selection decisions. Under the current system, leaf tissue is unavailable for breeders to sample for genotyping at the time of selection. Furthermore, breeding programs may evaluate tens of thousands of individual plants, making it unfeasible to maintain vegetative materials elsewhere for genotyping. For molecular breeding strategies like genomic selection (GS) or MAS in sweetpotato, parental and clonal genotypes must be available before December to utilize those data to make planting decisions and plan trials and nurseries. To generate genotypic data, tissues appropriate for DNA sampling must be available prior to December but after the previous seasons harvest. This timing allows for approximately 4 weeks for data analysis and breeding decisions, prior to the grafting of selected parental materials for winter greenhouse crossing nursery establishment in January. Typically, leaf tissue from greenhouse-sprouted storage roots is unavailable until March. A consequence of this is that genomic data is unavailable for, at best, five months after selections are made: November through March or April of the following year when sprouting occurs. Sweetpotato breeders must then make breeding decisions during a condensed period that coincides with increasingly busy field operations. As such, the current paradigm offsets the breeding cycle by a whole year: genome-based decisions are made after crossing nurseries are established, and selected parents must be grafted during the following season. Therefore, there is an urgent need for alternative and earlier genotyping options, particularly using storage root tissues which are available year-round in storage facilities. Currently, there is no literature describing the robust extraction and amplification of genomic DNA (gDNA) derived from sweetpotato storage root tissue. The methods proposed here provide options for acquiring high-quality gDNA, useful for DArTag and other genotyping platforms, from sweetpotato storage root tissues, substantially reducing resource waste while increasing workflow efficiencies.

## Materials and methods

### Plant materials, tissue collection, genotyping

#### Plant materials

Samples were collected from eight sweetpotato varieties or breeding lines maintained at North Carolina State University (NC State). These comprised a small but representative panel of breeding materials: orange-fleshed (‘Beauregard’, ‘Covington’, L14-31), white-fleshed (‘Murasaki-29’), yellow-fleshed (‘Norton’), and purple-fleshed (NCP06-0020, NCP16-0046, ‘Purple Majesty’).

Plant materials were maintained as vegetative vine cuttings and as storage roots; sampled tissues included leaf, storage root flesh (inner cambium), and storage root skin (periderm). All mentions of “root” tissues herein refer to storage root tissues. Flesh and skin tissues were considered because they are easy to collect and are readily available throughout the growing season and in off-season in storage facilities, providing a potential gDNA source that is available year-round. Vegetative plants were maintained at the Horticulture Field Lab (NC State, Raleigh, NC, USA) in a greenhouse with daily watering and biweekly fertilizer application (20-10-20) at 25–45 °C in 72-cell Landmark™ seedling trays (Stuewe & Sons, Corvallis, OR) in Fafard P4 soil mix (Fafard, Agawam, MA). Cured storage roots were collected from the sweetpotato storage facilities at Horticulture Crops Research Station (Clinton, NC, USA) which were maintained year-round at 15 °C and 85% relative humidity on a storage rack system providing ample airflow.

#### Tissue collection and preparation

All tissues were collected into a 1.1 mL Interlab MicroTube Rack System™ (TN0946-01R) on ice. Leaf tissue was collected in January of 2025, while storage roots were sampled from the 2024 harvest (approx. 4 months postharvest) and from the 2023 harvest (approx. 16 months postharvest) (Table 1). Leaf tissue was collected from the topmost fully expanded leaf on a vine; leaves were unfurled and tender. Selected leaves were folded in half, and two disks were collected simultaneously using a clean standard 6 mm office hole punch, avoiding ribs and dirty or damaged leaves. Leaf samples were lyophilized at 100mT and − 60 °C for 48 h (SP Scientific Benchtop Pro #BTP-9ESEVW) prior to sealing with silicone seals (MP53024).

**Table 1:** Sample design for sequencing quality and reproducibility

Year	Tissue	# Samples	Storage Age
2025	Leaf (Control)	8	0 month
2024	Periderm (Skin)	8	4 months
	Inner Cambium (Flesh)	8	
2023	Periderm (Skin)	8	16 months
	Inner Cambium (Flesh)	8	
Total		40	

**Fig. 1:**
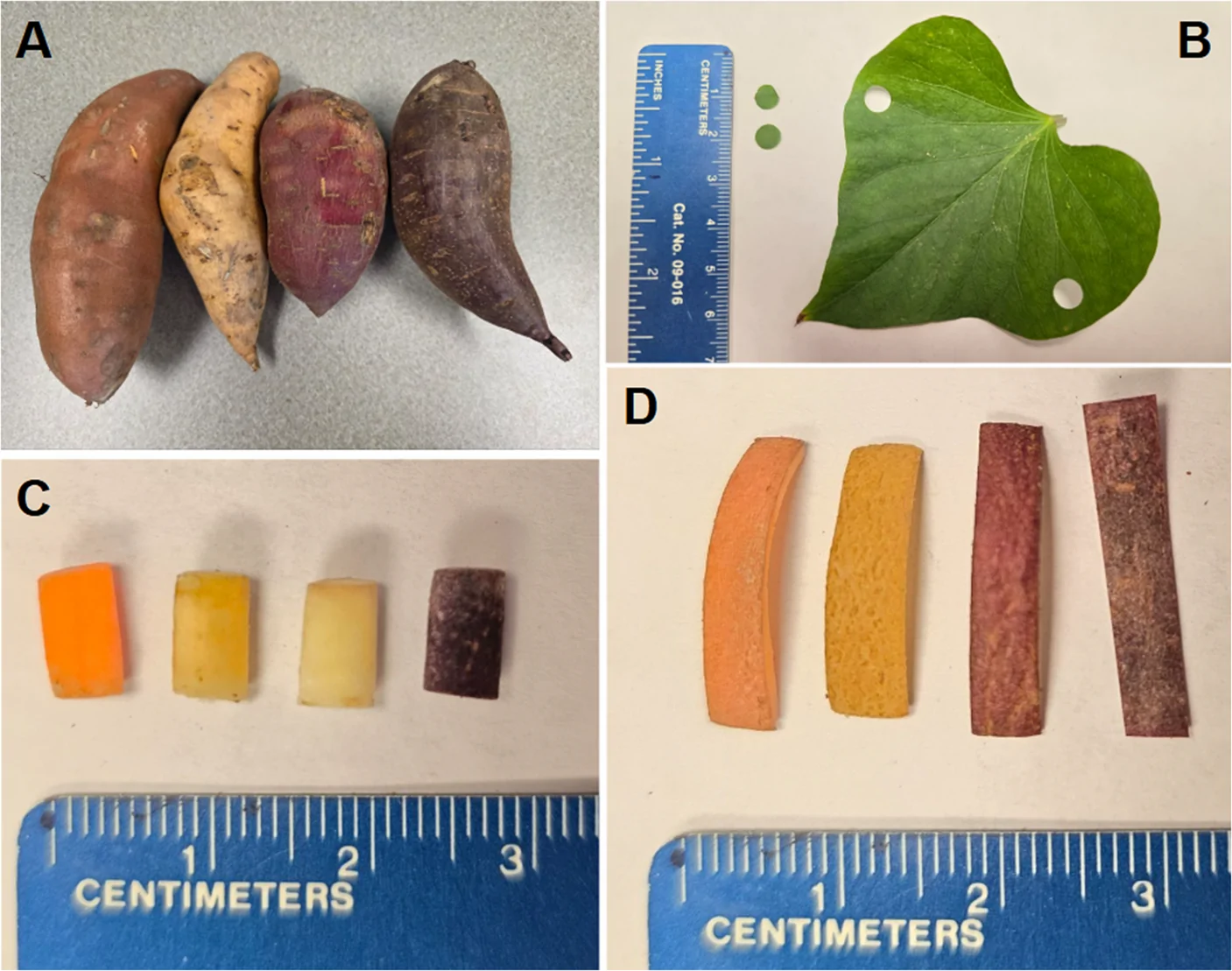
Sweetpotato tissue sample examples. **A** Sweetpotato storage roots; from left to right: ‘Covington’, ‘Norton’, ‘Murasaki-29’, and ‘Purple Majesty’. Tissue samples in C-D show tissues from these same roots, respectively. **B** Leaf tissue punches from a fully expanded leaf. **C** Cambium (flesh) tissue samples. **D** Periderm (skin) tissue samples

Prior to tissue sampling, storage roots were washed under tap water and gently scrubbed by hand to remove soil before air-drying at room temperature for 15 min. Next, using a vegetable peeler (Kuhn Rikon 2775, Switzerland), an approximately 1 mm layer of blemish-free skin tissue was removed. Using a sterile razor, a 4 mm x 20 mm strip of each skin sample (approx. 80mm^3^) was harvested into collection plates. Flesh tissue was collected using a sterile brass 4 mm diameter cork borer (United Scientific Supplies, CBST03), and the plug was ejected and trimmed using a razor to 7 mm in length (approx. 88mm^3^) before inserting into the sample tube. Root tissues were lyophilized for 96 h at 100mT and − 60 °C (Fig. 1). Prior to lyophilizing all tissue types, racks were covered with two layers of cheesecloth affixed with two rubber bands to prevent depressurization from agitating dried samples. After lyophilization, samples were macerated using 4 mm stainless steel grinding beads in a GenoGrinder (HG-400 MiniG^®^ 1600 Tissue Homogenizer and Cell Lyser, Metuchen, NJ) at 1500 RPM for 30 s intervals until completely pulverized. All flesh and skin samples weighed approximately 125 mg fresh weight. Sweetpotatoes typically range from 70 to 80% moisture in the NC State breeding program, and therefore dried root tissues provided approximately 25 mg per sample, which was the requested amount by Diversity Arrays Technology (DArT) for genotyping.

#### gDNA extraction and genotyping

The gDNA extraction was done by DArT (Diversity Arrays Technology Pty Ltd., Canberra, Australia). Four extraction methods were tested (Table 2) and only one yielded sufficient gDNA quantity and quality for downstream genomic analyses. The successful method 1 A used Macherey-Nagel (MN) Nucleomag kits (Macherey-Nagel GmbH & Co. KG, Düren, Germany) for gDNA extraction following the manufacturer’s protocol with modifications detailed as follows. Lyophilized and macerated sweetpotato tissue samples were processed in 1.1 mL microtubes (MTS-11-C-R-S, Corning Axygen, Union City, CA, USA) in 96-well rack format. To each sample, 600 µL of MN MC1 lysis buffer was added, and then tubes were shaken vigorously by hand to mix evenly, after which tubes were incubated at 60 °C for 2 h. Samples were then centrifuged for 10 min at 3,220 × *g* to separate the lysate. Next, 120 µL of the clear lysate was transferred to a microtiter plate (P-96–450 V Corning Axygen, Union City, CA, USA) and then 100 µL of MN MC2 binding buffer and MN NucleoMag c-Beads (at a ratio of 25:1) were added. DNA/bead separation, sequential washes with the MN Nucleomag kit buffers, and final elution in 30 µL EB buffer were performed using a Tecan 150 Freedom EVO automated liquid handling system (Tecan Trading AG, Männedorf, Switzerland). The quality and approximate assessment of gDNA quantity were measured via gel electrophoresis: for each sample, 5 µL aliquots were loaded onto a 0.8% 1×TAE agarose gel alongside a DNA ladder of known band size and DNA quantity, after which runs were conducted at 80 V for 30 min.

**Table 2:** Methodologies tested for sweetpotato gDNA extraction

Method	1		2	
Variant	A	B	A	B
**Lysis buffer**	Macherey-Nagel	Macherey-Nagel	AZORA	AZORA
**α-amylase**	no	yes	no	yes

Additional DNA extraction modifications were tested including transferring 110 µL of the clear lysate, adding 10 µL of α-amylase, and then vortexing and incubating the samples at 40 °C for 1 h (Methods 1 and 2, variant B); and replacing MN Lysis Buffer with the AZORA Biosciences Plant Lysis Buffer (Method 2 in both variants A and B, AZORA Biosciences, Sunshine Coast, Queensland, Australia).

Genotyping was performed by service provider DArT (Diversity Arrays Technology Pty Ltd., Canberra, Australia) using the product “SweetPotato_DArTag_BI_Cornell_University (1.0)” (3 K DArTag panel). All samples were genotyped across five replicate groups (the same eight cultivars per group): 2025 fresh leaf gDNA (*n* = 8), 2024 skin gDNA (*n* = 8), 2024 flesh gDNA (*n* = 8), 2023 skin gDNA (*n* = 8), and 2023 flesh gDNA (*n* = 8), totaling 40 samples (Table 1). The 3 K DArTag panel is composed of 3,120 genome-wide SNP markers and is described in Zhao et al. (2024). This panel was derived from whole genome skim sequencing data from a diverse set of 47 sweetpotato cultivars that represent the genetic diversity of North American sweetpotato breeding programs, and it has been validated across global sweetpotato populations. The 3 K DArTag panel is publicly available (Zhao et al. 2024).

### Marker filtering, dosage calling, sequencing performance, and comparative analyses

#### Marker filtering and dosage calling

The missing allele discovery count (MADC) file from DArT was used as raw genotyping data. It reports read counts at each of the marker loci for every sample, thereby capturing the allelic state at the target SNP together with any variants within the surrounding 81-bp region and thus multi-allelic information (Zhao et al. 2024). From the MADC file, microhaplotypes at each marker locus were classified into four categories: Ref, Alt, RefMatch, and AltMatch. Ref and Alt denote microhaplotypes carrying the reference and alternative allele at the target SNP with no additional variation at non-target positions in the amplicon. RefMatch and AltMatch carry the additional variants elsewhere in the 81-bp amplicon. Fixed microhaplotype IDs were assigned by HapApp (Zhao et al. 2026) to RefMatch and AltMatch for tracking and databasing, but downstream analyses used only Ref and Alt microhaplotypes and target SNPs. The following steps were then performed within the R Shiny application BIGapp (v1.5.1, RRID: SCR_026676- Sandercock et al. 2025) to obtain a VCF file with bi-allelic SNPs. First, read counts for the target SNPs were extracted from the MADC file and converted to a VCF file using the “Convert to VCF” module. Then, dosage calling was performed using the “Dosage Calling” module, with the R/updog package (v 2.1.5; Gerard et al. 2018) using the “norm” model and a ploidy of 6; SNPs that were missing for all samples (read depth = 0) were automatically removed by R/updog. The dosage-called VCF file was then filtered with the “VCF Filtering” module, so that any individual SNP call with a read depth less than 5 was assigned as missing (“NA”). This final step was necessary to account for R/updog’s default imputation method, which will assign a dosage value to sites when no reads are recorded. The resulting “low-stringency” marker set contained 2,485 SNPs and was used only for the initial sequencing-performance comparisons, including extraction-method, read-depth, and missing-data analyses. We applied only this limited filtering for these comparisons to avoid biasing the evaluation of extraction method performance by removing markers exhibiting high missingness and low depth, which were the metrics being assessed. We then generated a “high-stringency” marker set of 1,019 SNPs, which was used for downstream genetic comparisons, including dosage concordance, allele-frequency correlations, and PCA. These markers were filtered for high missing data (≥ 20%) and minor allele frequency (MAF) (≤ 0.05), ensuring a high-confidence dataset of informative markers shared across tissue samples for downstream evaluation.

#### Sequencing performance

We investigated (1) which DNA extraction method performed best across the evaluated tissue types, and (2) if comparable sequencing results can be obtained across tissue types and storage ages. For the initial performance assessment, means across samples were compared for missing data (defined as < 5 reads per marker per sample) and read depth. When determining the “best” DNA extraction method, we required the data to yield a mean read depth of at least 90 for each tissue type, which has been determined to be the minimum depth required for confident hexaploid dosage calling (Gerard et al. 2018). Following the determination of “best” extraction method, additional comparisons were made from these data using a more stringently filtered set to determine if downstream results were comparable across tissue types. Throughout these comparisons, we used the 2025 leaf tissue as the standard to which all other tissue and year types were compared. Since DNA extraction Method 1 A provided the highest mean read depth on average, with nearly 3x the performance of Method 1B and all tissue types exceeding 90 mean read depth (Fig. S1), we used the sequencing data from Method 1 A only for the downstream comparisons.

We compared sequencing performance between the leaf and root tissues through comparisons of read depth and missing data rates. To account for the repeated sampling of the different tissue types and storage years for the eight breeding lines, we used two statistical approaches to determine significance in R (v4.5.2; R Core Team 2025).

First, we determined if there were significant differences between leaf and root tissue age. For these tests, we used planned paired contrasts by breeding line, since leaf was collected for the year 2025 only. The read depth and missing data rates were compared between leaf and each root tissue age using Wilcoxon signed-rank tests by each breeding line with the wilcox.test function and paired=TRUE. To control for multiple testing, the resulting p-values were corrected using the R/stats p.adjust function and the Benjamini-Hochberg (false-discovery rate) method which provided the adjusted q-values (v4.5.2; R Core Team 2025). Significance was established for q-values less than 0.05.

Secondly, root tissues were evaluated to determine if sequencing performance differed by tissue type and storage duration. First, we estimated the effects of root tissue (skin vs. flesh) and storage age (4 vs. 16 months) on read depth using a linear mixed-effects model with the R/lme4 lmer() function (v1.1.37; Bates et al. 2015). Sequencing depth was log10-transformed (log10(DP + 1) and normality was improved. Missing rates, a ratio with values between 0 and 1, were evaluated with a generalized linear mixed model with a beta error distribution and logit link function (family = beta_family(link=”logit”) using the R/glmmTMB glmmTMB() function (v1.1.14; Brooks et al. 2017). In both models, root age, tissue type, and their interaction were set as fixed effects, while genotype was included as a random intercept to account for genetic variation. Both root age and tissue type were coded with sum-to-zero contrasts so that Type III tests correspond to main effects averaged over the other factor. Read depth significance was tested with a Type III ANOVA using the R/lmerTest method (v3.2.0; Kuznetsova et al. 2017), while missing rate significance was evaluated with a Type III Wald chi-square test using Anova() from the R/car package (v3.1.3; Fox and Weisberg 2019).

#### Comparative analyses

After evaluating sequencing performance for leaf and root tissues, we assessed whether downstream genetic signals were reproducible across tissues. Specifically, we compared sample-level genetic relationships using principal component analysis (PCA) and marker-level allele frequency estimates between tissue types. The goal was to determine if genotypic relationships and allele frequency between samples could be reproduced across the tissue types regardless of sequencing performance, which is particularly important for GS analysis.

For PCA, allele frequency comparisons, and dosage similarity analyses, additional filtering was applied to the 2,485 SNPs using the R/BIGr filterVCF() function (v0.6.2; RRID: SCR_026677; Sandercock et al. 2025). SNPs with ≥ 20% missing data were removed, and a MAF ≤ 0.05 filter was applied, resulting in 1,019 high-quality SNPs. For comparative analyses, all samples were retained regardless of missing data rates.

Dosage similarity was first compared pairwise between the root tissue ages and the leaf dosage values in two ways. First, exact dosage values were compared where the same individuals between tissue types had the exact dosage value as their counterpart across shared loci, divided by the total number of shared loci with non-missing dosage calls in both samples. Second, dosage values were compared with a ± 1 tolerance of the dosage values between the leaf and tested tissue type. Both methods were considered because hexaploid heterozygous calls may not be stable in individual SNP read depths less than 90 (Gerard et al. 2018), but should be approximately similar between individuals.

Allele frequencies were then compared to evaluate “population” summary metrics between tissue types across shared loci. Where highly similar allele frequencies were observed between tissue types, we inferred that downstream analysis that relied on allele frequencies would yield similar results. Matrices were subset for each tissue-age type and allele frequencies were calculated for the SNPs using the R/BIGr calculate_MAF() function (v0.6.2; RRID: SCR_026677; Sandercock et al. 2025). A linear regression was performed between the same SNPs from each tissue type, and the coefficient of determination (R^2^) was calculated, where a value greater than 0.95 was considered highly similar. To further evaluate agreement and potential systematic bias, regression slopes and intercepts were reported, and mean absolute error (MAE) was calculated as the mean absolute difference between root and leaf tissue derived allele frequencies across shared loci.

Finally, a principal component analysis (PCA) was performed to determine if (1) genetic relationships between individuals were maintained across tissue types, and (2) matching genotypes across tissue types occupied the same genetic space. This evaluated population structure across tissue types and storage years, regardless of any differences observed at the previous dosage comparison level. The 1,019 SNP dataset was first used to construct a VanRaden genomic relationship matrix using the R/AGHmatrix package with the Gmatrix() function (v3.0.1; Amadeu et al. 2023). This was followed by the base R prcomp(scale = TRUE) function to calculate the principal components from the relationship matrix. Individuals were visually compared for their locations in the resulting plots.

## Results

### DNA extraction and genotyping performance across tissues and storage ages

Both methods 1 A and 1B yielded good gDNA quality, however method 1B yielded suboptimal but still usable gDNA quantity. In both extraction methods, there were no observable discolored residues in any of the tissue types tested; these yellowish or brownish pigments can be indicative of phenolic compounds which can compromise gDNA quality and subsequent DArTag assay performance. For repeatability, if lysate discoloration is observed, the extract may require an additional round of purification prior to genotyping.

For the 3,120 SNPs on the DArTag 3 K panel, allele dosages were estimated from read counts. To limit low-coverage artifacts, any SNP-by-sample with fewer than five total reads were set to missing (NA). Markers showing complete missingness across all samples were removed, leaving 2,485 SNPs after minimal filtering. We then removed 1,377 monomorphic or near-monomorphic loci (MAF ≤ 5%) and applied a higher stringency filtering for missing rate (≥ 20%), resulting in a final set of 1,019 bi-allelic SNPs.

Leaf tissue had a significantly lower mean missing data rate (15.2%) when compared to all root tissue-age types (16.4% − 19.8%) (Table 3; q ≤ 0.039 for all pairwise comparisons). Consistent with the missing rate, the 2025 leaf tissue had a higher mean read depth (378.1) than all root tissue-age types (238.2–289.8). While the 2025 leaf tissue paired comparison to the 2023 skin tissue was not significant (q = 0.078), the remaining three paired comparisons for mean read depth were significant (q ≤ 0.031). All root tissue-age types obtained an optimal mean read depth greater than 90 (Table 3).

**Table 3:** Paired comparisons between leaf and root tissues at each storage age. Samples were compared for read depth and missing data rates to evaluate sequencing performance of different plant tissues. p-values were adjusted to q-values with the Benjamini-Hochberg (false discovery rate) to correct for multiple testing and were evaluated for significance (q < 0.05)

Tissue Type	Sample #	Storage Age (Month)	Mean Read Depth	Leaf Comparison (q-value)	Mean Missing Rate (%)	Leaf Comparison (q-value)
Leaf 2025	8	0	378.1	NA	15.2	NA
Flesh 2023	8	16	289.8	0.031	16.4	0.039
Flesh 2024	8	4	238.2	0.031	17.9	0.031
Skin 2023	8	16	251.9	0.078	19.8	0.039
Skin 2024	8	4	261.1	0.031	16.9	0.039

Detailed comparisons within root tissues revealed no significant effects of root tissue type on read depth (F(1,21) = 0.44, *p* = 0.51) or missing data (X^2^(1) = 0.57, *p* = 0.45). Similarly, storage age had no significant effect on read depth (F(1,21) = 0.02, *p* = 0.89) or missing data rates (X^2^(1) = 0.16, *p* = 0.69). Finally, there was no significant interaction between root tissue type and storage age for either metric (depth: F(1,21) = 2.02, *p* = 0.17; missing: X^2^(1) = 2.66, *p* = 0.1) (Supplementary Tables S2 and S3).

Dosage calls between samples showed high within-line concordance relative to the leaf reference (leaf 2025). Exact-dose matches averaged 85.5% (flesh 2023), 81.3% (flesh 2024), 82.8% (skin 2023), and 82.4% (skin 2024). Relaxing the threshold to a tolerance of ± 1 dose, a mean within-line concordance of > 97.3% was reached across all tissues and storage ages (Table 4).

**Table 4:** Within-line dosage concordance between each sweetpotato tissue and storage ages

Tissue	Comparison	Min	Max	Mean	SD
*Exact 1:1 Match to 2025 Leaf*					
Skin 2024	Exact Match	0.695	0.931	0.824	0.083
Flesh 2024	Exact Match	0.738	0.919	0.813	0.070
Skin 2023	Exact Match	0.637	0.945	0.828	0.109
Flesh 2023	Exact Match	0.765	0.928	0.855	0.057
*Tolerance Match (± 1) to 2025 Leaf*					
Skin 2024	Match (± 1)	0.950	1.000	0.976	0.018
Flesh 2024	Match (± 1)	0.936	0.998	0.974	0.022
Skin 2023	Match (± 1)	0.937	0.999	0.973	0.025
Flesh 2023	Match (± 1)	0.958	0.998	0.985	0.014

### Principal component analysis and allele frequencies

**Fig. 2:**
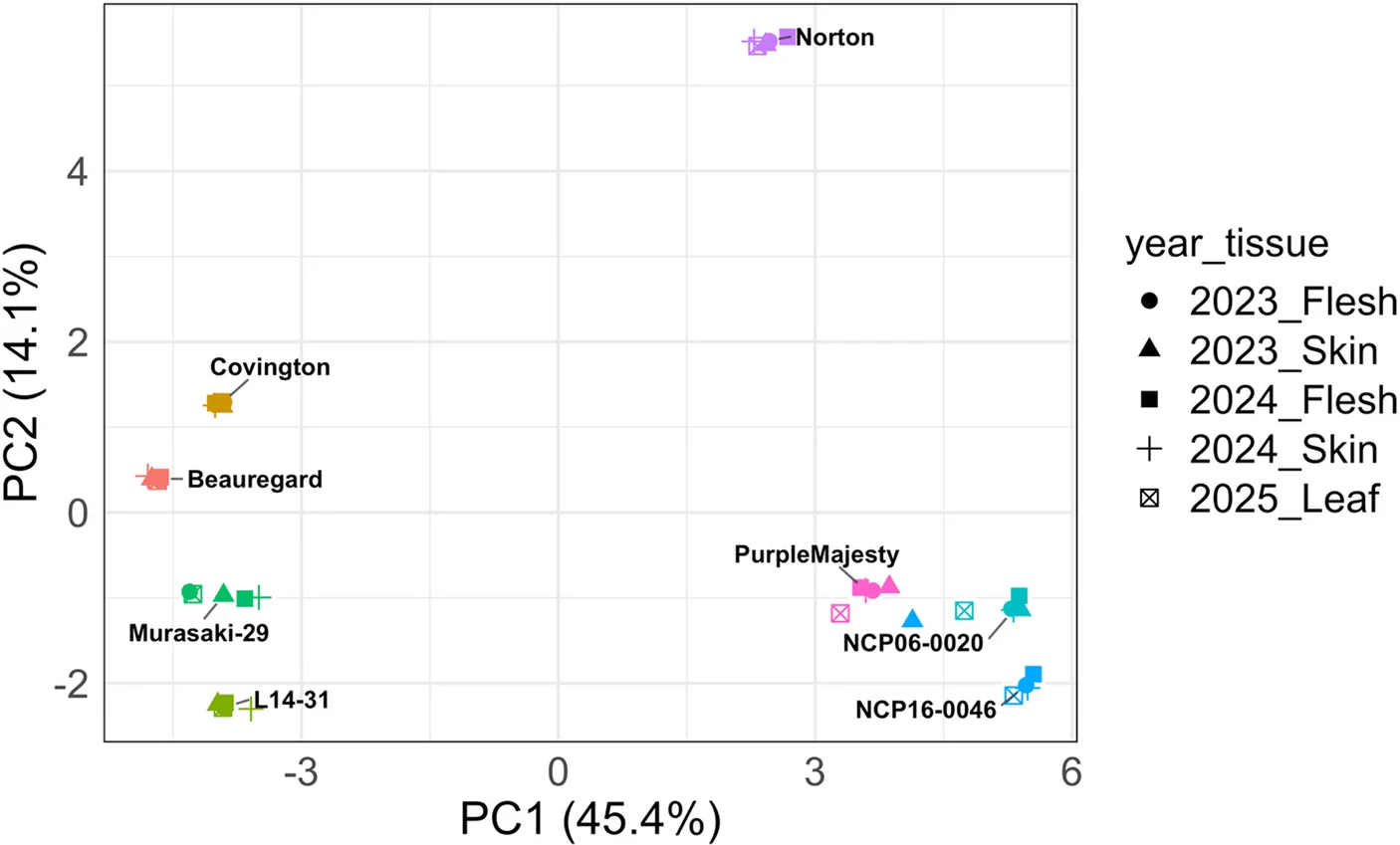
Principal component analysis evaluating the reproducibility of genotyped samples from each breeding line across tissue types and storage ages. Points on the plot represent genotyped samples, while the color corresponds to a given breeding line and shape represented the tissue-age type. Overall, breeding lines cluster tightly together regardless of tissue-age type. A single outlier from “NCP16-0046” was observed (marked with a red arrow)

PCA with the high-confidence set of 1,019 SNPs revealed that PC1 (45.4%), PC2 (14.1%), and PC3 (12.7%) described approximately 72.3% of genetic variance (Fig. 2). For all tissue types and storage ages, individuals showed high repeatability, clustering closely with themselves, with one exception where skin 2023 for NCP16-0046 was separate from the other NCP16-0046 tissue samples. Further inspection of this sample revealed poorer sequencing performance than the other NCP16-0046 samples, with a higher missing data rate (sample = 41.6%; breeding line = 22.3%) and lower mean read depth (sample = 23.8; breeding line = 222.5).

**Fig. 3:**
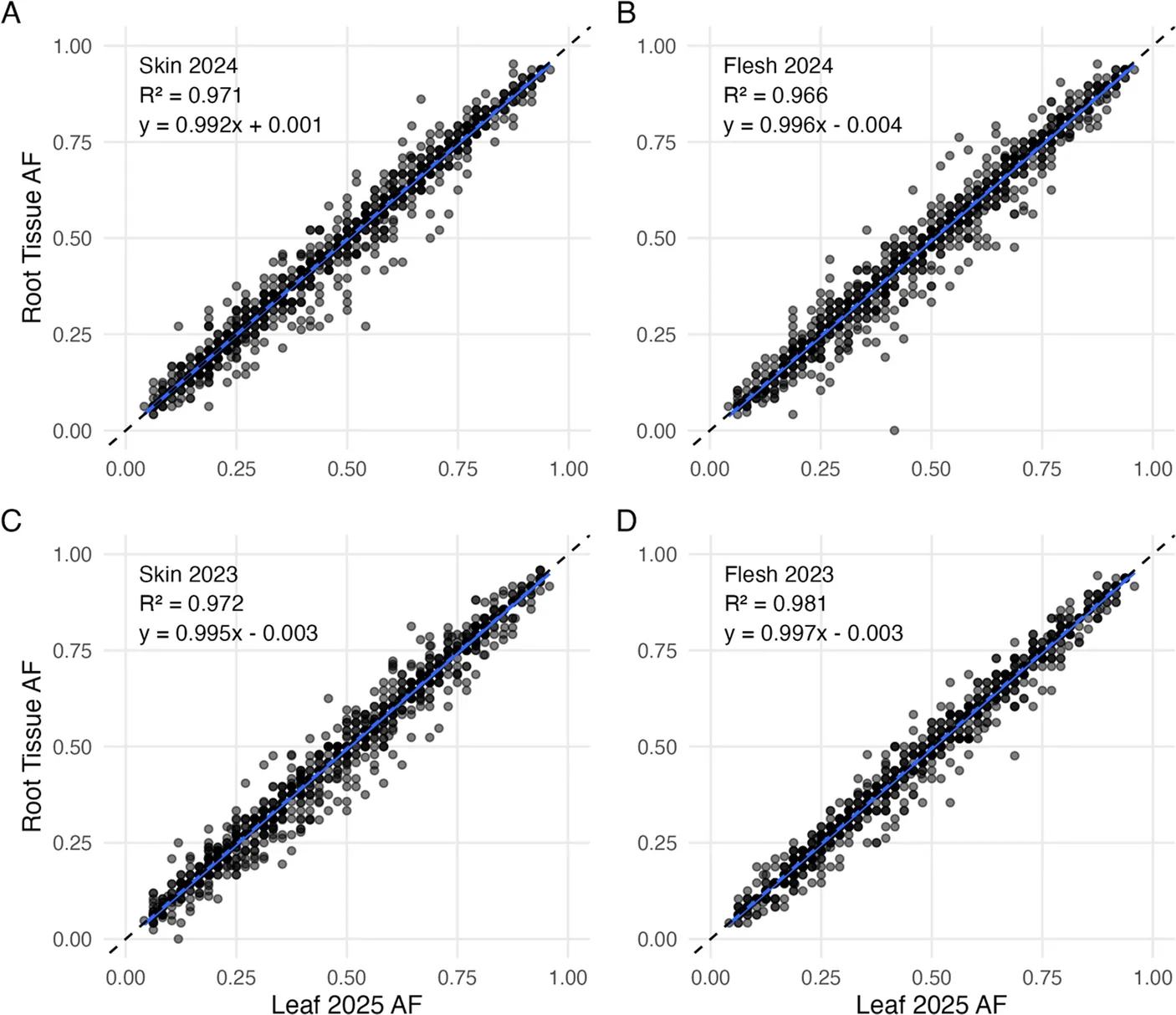
Allele frequency correlations across shared loci between 2025 leaf tissue and each storage root tissue type and storage age. The solid blue line is the linear regression of root allele frequency on leaf allele frequency. The dashed black line represents the ideal one-one agreement. The coefficient of determination (R^2^) and the regression equation are shown within each panel

Allele frequency correlations across shared loci were strong, with R^2^ > 0.96 for all pairwise comparisons of standard (leaf 2025) versus flesh and skin in 2023 and 2024 (Fig. 3). Regression slopes ranged from 0.992 to 0.997 and intercepts ranged from − 0.004 to 0.001 (Fig. 3). MAE values were 0.025 for 2024 skin, 0.028 for 2024 flesh, 0.027 for 2023 skin, and 0.020 for 2023 flesh, indicating close agreement with minimal systematic bias.

## Discussion

To the best of our knowledge, this is the first study comparing gDNA extracted from sweetpotato leaf and storage root tissues. Kim and Hamada (2005) described isolation of RNA from sweetpotato roots and gDNA from flowers, petioles, and stems, however, there does not appear to be any literature supporting gDNA extraction from the storage roots themselves. Their approach requires liquid nitrogen, which is an added expense, while our method used water ice. Puri et al. (2019) extracted DNA from storage roots, however, plant sequences (mitochondrial, plastid) were suppressed in order to perform a metagenomic analysis on root-associated microorganisms; the target for their research was not sweetpotato gDNA. The ability to extract gDNA from root tissue is critical for sweetpotato researchers, and breeders in particular, as genomic breeding strategies are increasingly being adopted and a rapid turnaround is needed to deploy these breeding strategies.

In comparing extracted DNA quality and read depth across tissue type and time, there was no major disadvantage to using storage root tissue as compared to leaf tissue. While leaf tissue had the highest mean read depth (378.09), storage root tissues were comparable, ranging from 238.17 at worst (2024 flesh) to 289.79 (2023 flesh) at best. Additionally, a previous study determined that a minimum read depth of 90 is sufficient for accurately (> 90%) estimating the correct genotype dosage value (Gerard et al. 2018), thus all storage root tissues and ages evaluated exceeded the minimum mean threshold for accurate dosage calling performance. Similarly, while leaf tissue had the lowest mean missing data (15.2%), root tissues ranged from 19.8% (2023 skin) to 16.4% (2023 flesh). Older roots genotyped just as well, and, across years, all tissue types yielded similar gDNA extractions. Root and leaf tissues provided similar genotyping quality, and between root tissue types, a slight edge was observed in storage root flesh over skin, though it was not significant. The single NCP16-0046 skin sample from 2023 that appeared as a separated outlier in the PCA had poorer sequencing performance than all other samples, with 41.6% missing data and 23.8 mean read depth. These data quality values suggest its separation from the other tightly clustered NCP16-0046 tissue samples was driven by reduced sequencing performance, potentially from tissue or DNA quality, and not biological differentiation.

For downstream comparative analyses, we used a high-stringency set of 1,019 SNPs, which was a large reduction from the low-stringency set of 2,485 SNPs used when comparing the Method 1 A and Method 1B DNA extraction methods. Of the 1,466 markers removed during filtering, 1,377 (94%) were monomorphic or near-monomorphic across all samples (MAF ≤ 5%). Retaining these non-informative SNPs would artificially inflate the dosage concordance estimates without testing the reproducibility of the different hexaploid dosage calls at polymorphic sites. Additionally, these non-informative SNPs are typically removed during routine breeding analyses such as GWAS and GS. The remaining 89 SNPs were removed due to greater than 20% missing data, which ensured each comparative analysis used a jointly filtered SNP set that was shared across most samples.

With regard to the PCA, ‘Beauregard’ (Rolston et al. 1987), ‘Covington’ (Yencho et al. 2008), ‘Murasaki-29’ (LaBonte et al. 2008), and L14-31 (2014, unreleased) all originate from NC State or LSU within the last 40 years, and are expected to have significant overlap in genomic relatedness. Similarly, the purple clones NCP06-0020 (2006, unreleased), NCP16-0046 (2016, unreleased), and ‘Purple Majesty’ (Yencho and Pecota 2022) all originate from NC State in the last 20 years and share common ancestry. ‘Norton’, a standalone heirloom variety as depicted in the PCA, is a US Civil War era cultivar, and is not closely related to any of the other lines evaluated (Morgan and Ross 1892).

The eight cultivars genotyped in this study represent a somewhat limited panel in sweetpotato diversity. However, Zhao et al. (2026) applied this 3 K DArTag panel to samples from breeding programs originating in Africa, Asia, Central America/Caribbean, North America, and South America. Population genomic analyses with 4,087 samples established the utility of the genotyping panel across geographically diverse sweetpotato breeding materials, but it did not compare DNA extraction and genotyping performance between leaf and root tissue sources. Thus, root tissue DNA extraction and genotyping performance may vary across wild relatives, landraces, or in particular, high starch and high phenolic lines. Evaluation across a broader germplasm panel will therefore be needed to establish the general applicability of the protocol.

Because tissue types performed similarly, we do not expect that leaf, skin, or flesh tissue type had significant barriers to gDNA extraction using the described methods. Kumar et al. (2007) described challenges in RNA extraction from sweetpotato roots due to polysaccharides, starches, polyphenols, and more, which we expected to be similar issues for DNA extraction. Sweetpotato storage roots also contain sugars, latex, carotenoids and/or anthocyanins, and other compounds that are less abundant or absent in the leaves, and these too could have interfered with DNA extraction. While prior literature suggests challenges in nucleic acid extraction from root tissues, our methods were not significantly impacted by these. In our methodologies, one possible reason for greater performance by flesh over skin tissue is due to the composition of skin, which contains an outer peridermal cell layer that lignifies and is sloughed during storage root growth (Firon et al. 2009). This lignified “dead” tissue may, in part, explain the slightly reduced DNA quality, though differences were minor. Other possible reasons for reduced DNA quality could be due to environmental contaminants like soil and microorganisms, or latex exuded from the storage root itself. Leaf tissue, being much thinner, lyophilized much faster. As such this may remain the preferred tissue type for silica or air-drying methods. However, in the protocols described, all three tissue types are sufficient for gDNA extraction for DArTag genotyping.

In addition to DArTag and other current genotyping approaches, next-generation approaches may likewise benefit from gDNA extracted from storage root tissues, although protocols will need to be explored. Whole genome sequencing, ultra-high density genotyping, and others may benefit from the year-round availability of sweetpotato storage root tissues, provided the extracted DNA meets the high-quality standards needed for data generation in these applications. Metagenomic and transcriptomic studies might also be considered, which may show differential expression between tissue types. Ultimately, sweetpotato storage roots provide a suitable source for gDNA, providing a broader window for breeders and other scientists to use molecular approaches for faster, more efficient variety development.

## Electronic Supplementary Material

Below is the link to the electronic supplementary material.

Supplementary Materials

## Data Availability

Genomic data from the described materials is available from the corresponding author upon request. The sequencing metrics for each genotyped sample are included as a CSV, available on Zenodo at https://doi.org/10.5281/zenodo.22288064.

## Abbreviations

AFLP: amplified fragment length polymorphism
DArT: Diversity Array Technologies
DArTag: Diversity Array Technologies targeted amplicon-based genotyping
DNA: deoxyribonucleic acid
gDNA: genomic deoxyribonucleic acid
GS: genomic selection
MADC: missing allele discovery count
MAF: minor allele frequency
MAS: marker-assisted selection
PCA: principal component analysis
RH: relative humidity
RPM: revolutions per minute
SNP: single nucleotide polymorphism
SSR: simple sequence repeat
