# Supplementary material for: DNA extraction from sweetpotato (Ipomoea batatas) root tissues supports routine genotyping
Source: Mol Breed. 2026 Sep 19;46(10):95. doi: 10.1007/s11032-026-01718-w (PMC13589723; doi:10.1007/s11032-026-01718-w)
Supplement: Supplementary file 1 [file 11032_2026_1718_MOESM1_ESM.docx]

**Supplementary Figures and Tables**

**Table S1.** Number of non-missing SNPs present in each tissue-age group across different presence thresholds.

| **% Present in Sample** | **#2025 Leaf SNPs** | **#2024 Skin SNPs** | **#2024 Flesh SNPs** | **#2023 Skin SNPs** | **#2023 Flesh SNPs** |
| --- | --- | --- | --- | --- | --- |
| ≥0% | 2485 | 2485 | 2485 | 2485 | 2485 |
| ≥10% | 2468 | 2452 | 2443 | 2446 | 2452 |
| ≥20% | 2367 | 2358 | 2325 | 2332 | 2348 |
| ≥30% | 2264 | 2241 | 2216 | 2219 | 2241 |
| ≥40% | 2191 | 2162 | 2132 | 2132 | 2163 |
| ≥50% | 2191 | 2162 | 2132 | 2132 | 2163 |
| ≥60% | 2097 | 2058 | 2033 | 2032 | 2071 |
| ≥70% | 1983 | 1942 | 1916 | 1898 | 1952 |
| ≥80% | 1863 | 1802 | 1780 | 1702 | 1816 |
| ≥90% | 1617 | 1507 | 1476 | 1183 | 1568 |
| 100% | 1617 | 1507 | 1476 | 1183 | 1568 |

**Table S2.** Type III Analysis of Variance Table with Satterthwaite's method for the log10 of mean read depth from the linear mixed-effects model.

|  | **Sum Sq** | **Mean Sq** | **NumDF** | **DenDF** | **F value** | **Pr(>F)** |
| --- | --- | --- | --- | --- | --- | --- |
| RootAge | 0.000891 | 0.000891 | 1 | 21 | 0.0211 | 0.8858 |
| RootTissue | 0.018638 | 0.018638 | 1 | 21 | 0.4421 | 0.5133 |
| RootAge:RootTissue | 0.085016 | 0.085016 | 1 | 21 | 2.0168 | 0.1702 |

**Table S3.** Analysis of Deviance Table (Type III Wald chi-square tests) for the mean missing rate from the generalized linear mixed effects model.

|  | **Chisq** | **Df** | **Pr(>X^2^)** |
| --- | --- | --- | --- |
| (Intercept) | 507.9749 | 1 | < 2e-16 |
| RootAge | 0.1560 | 1 | 0.6929 |
| RootTissue | 0.5667 | 1 | 0.4516 |
| RootAge:RootTissue | 2.6551 | 1 | 0.10322 |

**Fig. S1** Mean read depth distributions for each DNA extraction method. The dashed vertical lines are the means for each extraction method (Method 1A mean = 283.8; Method 1B mean = 95.1).
